# Graham’s Chemistry

**Published:** 1852-05

**Authors:** 


### GRAHAM'S CHEMISTRY.

A new edition of this able work is issuing from the press of Blanchard & Lea, to which the American profession is so largely indebted. One half has appeared and forms a volume of 430 pages. No English chemist ranks higher than Prof. Graham, and his work is one of the most lucid and philosophical that can be placed in the hands of a student. The publishers are doing their part well; they are sending it forth in the best style. Every physician, at least every physician who receives students into his office, must occasionally refresh his memory in this progressive science, and we could not refer them to any work in our language superior to Graham's chemistry.

